# Efficacy and safety of DL‐3‐n‐butylphthalide in patients with mild cognitive impairment: a multicentre, randomized, double‐blind, placebo‐controlled trial

**DOI:** 10.1002/alz.088925

**Published:** 2025-01-09

**Authors:** Pin Wang, Wenxian Sun, Jin Gong, Xiaodong Han, Chang Xu, Yufei Chen, Yuting Yang, Heya Luan, Shaoqi Li, Ruina Li, Boye Wen, Sirong Lv, Cuibai Wei

**Affiliations:** ^1^ Xuanwu Hospital, Capital Medical University, Beijing, Beijing China; ^2^ Innovation Center for Neurological Disorders and Department of Neurology, Xuanwu Hospital, Capital Medical University, Beijing, Beijing China; ^3^ School of Biological Science and Medical Engineering, Beihang University, Beijing, Beijing China

## Abstract

**Background:**

The DL‐3‐n‐butylphthalide (NBP), a multi‐target neuroprotective drug, improving cognitive impairment in patient with vascular cognitive impairment has been confirmed. The efficacy of NBP in patients with cognitive impairment due to Alzheimer’s disease (AD) remains unknown. This study aimed to evaluate the efficacy and safety of NBP in patients with mild cognitive impairment (MCI) due to AD though a clinical randomized controlled trail.

**Method:**

This is a 12‐month, randomized, double‐blind, placebo‐controlled, multicentric trial. The trial included patients 50 to 85 years of age who had MCI due to AD, with a clinical dementia rating (CDR) score 0.5 and a mini‐mental state examination (MMSE) score 20‐26. Subjects were randomly assigned to receive either NBP soft capsule (200mg, three times a day), or equivalent dose of placebo for up to 12 months with an allocation ratio of 1:1. The primary endpoint is the change in 12‐item Alzheimer’s disease assessment scale‐cognitive subscale (ADAS‐cog_12_; range, from 0 to 75, with lower scores indicating lesser severity) after 12 months. Secondary outcomes included the change in scores on the MMSE, the Sum of Boxes of CDR (CDR‐SB), Activities of Daily Living (ADL), Neuropsychiatric inventory (NPI), Auditory Verbal Learning Test (AVLT), Trail Making Test (TMT), Digit Span, as well as the standardized uptake ratio (SUVR) on FDG‐PET. All patients were monitored for adverse events (AEs).

**Result:**

A total of 270 patients were enrolled, with 135 assigned to receive NBP soft capsule and 135 to receive placebo. The mean ADAS‐cog score at baseline was approximately 12 in both groups. The change from baseline in the ADAS‐cog score at 12 months was ‐1.30 with NBP and 0.73 with placebo (difference, ‐2.04 95% confidence interval [CI], ‐2.92 to ‐1.16; P = 0.0001). Other mean differences between the two groups in the change from baseline favoring BNP were Immediate Recall score, 1.53, (95% CI, 0.47 to 2.59; P = 0.0111). NBP‐related AE were uncommon and primarily consisted of liver dysfunction and nausea symptoms.

**Conclusion:**

The results of the 12‐month treatment period showed that NBP was effective in improving cognitive function in patients with MCI due to AD and exhibited good safety.

